# The characteristics of pediatric emergency department visits in Korea: An observational study analyzing Korea Health Panel data

**DOI:** 10.1371/journal.pone.0197929

**Published:** 2018-05-24

**Authors:** Dong Hyun Seo, Min Joung Kim, Kyung Hwan Kim, Junseok Park, Dong Wun Shin, Hoon Kim, Woochan Jeon, Hyunjong Kim, Joon Min Park

**Affiliations:** 1 Department of Emergency Medicine, Inje University Ilsan Paik Hospital, Goyang, Korea; 2 Department of Emergency Medicine, Yonsei University College of Medicine, Seoul, Korea; Medical University Graz, AUSTRIA

## Abstract

**Objective:**

We investigated the characteristics of pediatric emergency department (ED) patients in Korea and determined factors associated with hospital admission after ED treatment.

**Methods:**

Korea Health Panel data from 2008 through 2013 were analyzed retrospectively; we included patients under 18 years old who visited the ED at least once. We collected patient and household epidemiologic data such as sex, age group, region of residence, disability, chronic disease, household income quintile, national health insurance type, use of private insurance, and annual frequency of ED visits. We also examined data related to each ED visit, such as reason for visit, medical service provided, and hospital size/ownership. We then investigated which factors were correlated with case disposition (discharge home or hospital admission) after ED treatment.

**Results:**

In total, 3,160 pediatric ED visits occurred during the six-year period. Males (57.5%) and children aged 0–5 years (47.7%) made more visits than females and older children, respectively. The proportion of ED visits for disease (67.7%) was much higher than for injury or poisoning (32.2%), and 452 cases (14.3%) required hospital admission. For hospital admission, the odds ratio (OR) of females was 0.73 compared to males, and the OR of children aged 6–11 was 0.68 compared to children aged 0–5. The OR of capital residents was 0.69 compared to province residents, and the OR of the highest income quintile was 0.51 compared to the lowest quintile. The OR of children with private insurance coverage was 0.49 compared to those lacking private insurance, and the OR of ED visits due to disease was 1.82 compared to visits due to injury/poisoning.

**Conclusion:**

This analysis of clinical and demographic characteristics of pediatric ED visits and hospital admissions can serve as the foundation of future prospective studies required for establishing appropriate policies for the Korean pediatric emergency medical system.

## Introduction

Patient visits to the emergency department (ED) have been increasing worldwide for several decades [1,2]. Although the precise reasons are not known, changes in transportation, increased leisure time, and improved access to medical service could be factors. Pediatric patients account for a significant portion of ED visits (31.2% in Korea and 20.5% in the US) [3,4]. Many ED patients visit EDs for illnesses or injuries that do not require emergency treatment, or for conditions that are treatable or preventable by primary care [5,6], and previous studies reported that 58% to 82% of all pediatric patients visited the ED with non-urgent conditions [7,8]. Possible reasons might be the lack of appropriate alternative medical facilities, convenience of ED access, or overestimation of the severity of children’s symptoms by parents [9,10].

The increase in absolute number of ED visits and proportion of non-urgent visits are the main factors affecting ED overcrowding [11]. Overcrowding is problematic in that it can impede appropriate and timely treatment due to diversion of limited resources, resulting in poor prognoses and negatively affecting working staff and patient satisfaction [12]. To resolve ED overcrowding, it is important to first identify the characteristics of ED visits, and based on this, intervention strategies could be explored.

In Korea, patients can use ED services directly whenever necessary without a prior primary clinic visit, regardless of the acuity or severity of illness. In 2016, 539 EDs were available around the country. With regard to the characteristics of pediatric ED visits in Korea, Kwak et al. [3] reported an analysis of nationwide data from the National Emergency Department Information System (NEDIS) of Korea. That study primarily investigated the clinical characteristics of patients at the time of the ED visit. However, in addition to patients’ clinical characteristics, demographic factors also affect use of ED services. Therefore, a comprehensive analysis of pediatric ED patients investigating both clinical and demographic characteristics can provide baseline data for establishing desirable policies for pediatric EDs. In this study, we investigated characteristics of pediatric ED patients and their families, and explored characteristics related to hospital admission by analyzing nationwide panel data in Korea.

## Methods

After approval of this study by the ethics committee of Inje Univsersity Ilsan Paik Hospital (Goyang, Korea), we analyzed Korea Health Panel Study (KHPS) data (version 1.2.2) for a six-year period (2008–2013). Due to the retrospective nature of our study, the requirement for informed consent was waived. In addition, we obtained KHPS data that were fully anonymized before analysis.

### Survey data source

The KHPS is an official statistical investigation and has been conducted annually since 2008 under the supervision of the Korea Institute for Health and Social Affairs and the National Health Insurance Service. The KHPS aims to acquire baseline data for establishing and improving health care policies and health insurance policies by examining data related to medical service use, medical expenditures, and insurance coverage. The sampling frame was set to 90% of the national population surveyed in the 2005 Population Census of Korea, and a two-stage probability proportionate and stratified cluster sampling method was used. First, the population was stratified according to geographic area using household registries (16 metropolitan cities and provinces, and two towns [*dongs* and *eups/myeons*]); this yielded a total of 237,165 clusters. From the whole population clusters, 350 sample clusters were extracted, and then sample households were extracted from those clusters. Finally, family members from these sample households became the Korean Health Panel [13]. At the start, about 8,000 households across the nation were sampled, and the survey was conducted once or twice a year using both self-reporting questionnaires and in-person interviews with trained investigators. Response rates during each year of the survey are shown in Table 1. Receipts for medical expenses and prescriptions were used as supporting information about use of medical services by household members. Most of the children’s data were collected by their parents, and most surveys were conducted within one year of each ED visit. However, the exact time gap between the ED visit and survey completion was not fixed.

**Table 1:** KHPS survey response rates.

Year	Households	Subjects	
	N	N	(% of original subjects)
2008 (first wave)	7,866	24,616	100.0
2008 (second wave)	7,201	22,594	91.8
2009	6,798	21,125	85.8
2010 (first wave)	6,433	19,841	80.6
2010 (second wave)	6,283	19,163	77.8
2011	6,041	18,257	74.2
2012	5,850	17,414	70.7
2013	7,743	22,701	-*

*Between the 2012 and 2013 surveys, 6,454 subjects from 2,222 households were newly enrolled in the panel.

### Subjects

From the panel, we included children under the age of 18 who had visited the ED at least once between 2008 and 2013. When a child visited the ED more than once a year, each visit was considered as an independent case.

### Data collection and statistical analysis

First, we collected demographic data related to each pediatric ED patient: sex, age, region of residence, presence of disability, presence of chronic illness (defined as a continuation of the same symptoms for more than three months), household income, national health insurance service type, presence of private insurance, and frequency of annual ED visits (visits within a calendar year). Next, we gathered information regarding ED service: mode of transport to the ED, reason for the visit, medical services provided, and disposition (Result) of the case. Lastly, we determined the ownership and size of hospital.

Before conducting statistical analyses, we categorized the variables as follows. Children were grouped into three age ranges (0–5, 6–11, and 12–17 years); region of residence was divided into three groups (capital, metropolitan city, and province). Income was classified in quintiles (the fifth quintile as the highest household income). The frequency of annual ED visits was categorized as single or multiple; transport method was categorized as self-transport or ambulance. Hospitals were categorized into three size groups (tertiary hospital, general hospital, and hospital/clinic); hospital ownership was classified as public or private. Case dispositions were categorized as discharged home, admitted, or transferred to another hospital. The latter two groups were combined into an “admission” group for some analyses.

We calculated frequency and percentage for each variable, and evaluated differences between groups using the chi-square test or Fisher’s exact test. We performed multivariate logistic regression analysis with disposition as the dependent variable, and used the forward conditional selection method to determine the independent variables. Statistical analysis was performed using SPSS Statistics for Windows version 21 (IBM, Armonk, NY, USA), and a p-value <0.05 was considered to be statistically significant.

## Results

### Overall characteristics

A total of 3,160 pediatric ED visits occurred during the study period. Table 2 shows that the proportion of male children (57.5%) was higher than that of females, and of the age groups, those aged 0–5 years visited EDs the most (47.7%). For residence region, province ranked highest, followed by metropolitan city and capital. For household income, the fourth quintile produced the most visits, followed by the third and first quintiles. Among all children, 87.4% were covered by private health insurance, and 40.5% visited the ED more than twice a year. Transportation via ambulance accounted for only 5.2% of visits, and disease as the reason for the ED visit was more than twice as common as injury/poisoning as the reason. Among all the medical services provided in the ED, examination without medication or surgical intervention was most common (88.3%). In terms of size, visits to general hospitals were most common (53.2%). Public hospitals accounted for only 7.8% of visits. Only 14.3% of children required hospital admission or transfer to another hospital as a result of the ED visit.

**Table 2:** Case characteristics during study period.

Variable	N	(%)
Sex		
Male	1816	(57.5)
Female	1344	(42.5)
Age (years)		
0–5	1507	(47.7)
6–11	935	(29.6)
12–17	718	(22.7)
Region		
Province	1868	(59.1)
Metropolitan city	802	(25.4)
Capital	490	(15.5)
Household income		
Quintile 1	235	(7.4)
Quintile 2	547	(17.3)
Quintile 3	809	(25.6)
Quintile 4	890	(28.2)
Quintile 5	665	(21.0)
Unspecified	14	(0.4)
Handicap		
No	3123	(98.8)
Yes	37	(1.2)
Chronic disease		
No	2232	(70.6)
Yes	928	(29.4)
National health insurance		
NHI	2975	(94.1)
Medicaid	185	(5.9)
Private insurance		
No	271	(8.6)
Yes	2763	(87.4)
Unspecified	126	(4.0)
Annual ED visits		
Single	1881	(59.5)
Multiple	1279	(40.5)
Transport method		
Self	2996	(94.8)
Ambulance	164	(5.2)
Reason for visit		
Injury/poison	1017	(32.2)
Disease	2138	(67.7)
Other	5	(0.2)
Provided medical service		
Examination (only)	2791	(88.3)
Emergency medication/procedure	188	(5.9)
Operation	168	(5.3)
Unspecified	13	(0.4)
Hospital size		
Hospital/clinic	869	(27.5)
General hospital	1680	(53.2)
Tertiary hospital	611	(19.3)
Hospital ownership		
Public	245	(7.8)
Private	2915	(92.2)
Disposition		
Home discharge	2708	(85.7)
Transfer	65	(2.1)
Admission	387	(12.2)

### Discharge group versus admission group

Table 3 shows that male children were more likely than females to require hospital admission, and children aged 6–11 years formed a smaller proportion of admissions than did the other age groups. The proportion of admissions was larger in the first and second household income quintiles than in the fourth and fifth quintiles. Medicaid beneficiaries were more likely to be admitted than National Health Insurance (NHI) beneficiaries, as were those not covered by private insurance. ED visits via ambulance and due to disease formed larger proportions of the admission group than those via self-transportation and due to injury/poisoning, respectively. Patients requiring a surgical operation were more likely to be admitted than those receiving other ED services.

**Table 3:** Characteristics of discharge group and transfer/admission group.

Variable	Discharge		Admission		
	N	(%)	N	(%)	p-value
Gender					
Male	1528	(84.1)	288	(15.9)	0.004
Female	1180	(87.8)	164	(12.2)	
Age (years)					
0–5	1277	(84.7)	230	(15.3)	<0.001
6–11	836	(89.4)	99	(10.6)	
12–17	595	(82.9)	123	(17.1)	
Region					
Province	1580	(84.6)	288	(15.4)	0.049
Metropolitan city	693	(86.4)	109	(13.6)	
Capital	435	(88.8)	55	(11.2)	
Household income					
Quintile 1	191	(81.3)	44	(18.7)	<0.001
Quintile 2	438	(80.1)	109	(19.9)	
Quintile 3	677	(83.7)	132	(16.3)	
Quintile 4	786	(88.3)	104	(11.7)	
Quintile 5	605	(91.0)	60	(9.0)	
Unspecified	11	(78.6)	3	(21.4)	
Handicap					
No	2679	(85.8)	444	(14.2)	0.201
Yes	29	(78.4)	8	(21.6)	
Chronic disease					
No	1913	(85.7)	319	(14.3)	0.977
Yes	795	(85.7)	133	(14.3)	
National health insurance					
NHI	2566	(86.3)	409	(13.7)	<0.001
Medicaid	142	(76.8)	43	(23.2)	
Private insurance					
No	205	(75.6)	66	(24.4)	<0.001
Yes	2397	(86.8)	366	(13.2)	
Unspecified	106	(84.1)	20	(15.9)	
Annual ED visits					
Single	1641	(87.2)	240	(12.8)	0.003
Multiple	1067	(83.4)	212	(16.6)	
Transport method					
Self	2591	(86.5)	405	(13.5)	<0.001
Ambulance	117	(71.3)	47	(28.7)	
Reason for visit*					
Injury/poisoning	902	(88.7)	115	(11.3)	0.003
Disease	1801	(84.2)	337	(15.8)	
Other	5	(100)	0	(0)	
Provided medical service					
Examination (only)	2429	(87.0)	362	(13.0)	<0.001
Emergency medication/procedure	165	(87.8)	23	(12.2)	
Operation	107	(63.7)	61	(36.3)	
Unspecified	7	(53.8)	6	(46.2)	
Hospital type					
Hospital/clinic	753	(86.7)	116	(13.3)	0.629
General hospital	1435	(85.4)	245	(14.6)	
Tertiary hospital	520	(85.1)	91	(14.9)	
Hospital ownership					
Public	207	(84.5)	38	(15.5)	0.569
Private	2501	(85.8)	414	(14.2)	

*Fisher’s exact test was used.

### Factors associated with hospital admission

Table 4 shows that variables significantly associated with hospital admission included sex, age, region, private insurance coverage, frequency of ED visits, transport method, reason for visit, and medical services provided. Females exhibited an odds ratio (OR) of 0.73 compared with males; children aged 6–11 years showed an OR of 0.68 compared with those aged 0–5. Residents in provinces had an OR of 0.69 compared with those in the capital, and children in the fifth quintile of household income had an OR of 0.51 compared with the first quintile. Privately insured children had an OR of 0.49 compared to those lacking private insurance, and children with multiple annual ED visits had an OR of 1.33 compared to those with a single annual ED visit. The OR for ambulance transport was 2.98 compared with self-transport, and patients visiting the ED due to disease had an OR of 1.82 compared with those visiting dues to injury/poisoning. Patients who underwent a surgical operation had higher odds than those who received only a diagnostic examination (OR = 5.4).

**Table 4:** Factors associated with transfer/admission and their odds ratio.

Variable	Unadjusted			Adjusted		
	OR	95% CI	p-value	OR	95% CI	p-value
Sex						
Male	Reference			Reference		
Female	0.74	0.60–0.91	<0.001	0.73	0.59–0.92	0.006
Age (years)						
0–5	Reference			Reference		
6–11	0.66	0.51–0.85	.001	0.68	0.52–0.89	0.006
12–17	1.15	0.90–1.46	.260	1.16	0.89–1.52	0.280
Region						
Province	Reference			Reference		
Metropolitan city	0.86	0.68–1.10	0.224	0.81	0.62–1.04	0.099
Capital	0.69	0.51–0.94	0.020	0.69	0.49–0.96	0.026
Household income						
Quintile 1	Reference			Reference		
Quintile 2	1.08	0.73–1.59	0.697	1.26	0.82–1.94	0.294
Quintile 3	0.85	0.58–1.23	0.386	0.98	0.64–1.50	0.917
Quintile 4	0.57	0.39–0.85	0.005	0.72	0.47–1.11	0.138
Quintile 5	0.43	0.28–0.66	<0.001	0.51	0.32–0.82	0.005
Private insurance						
No	Reference			Reference		
Yes	0.47	0.35–0.64	<0.001	0.49	0.35–0.67	<0.001
Annual ED visits						
Single	Reference			Reference		
Multiple	1.36	1.11–1.66	0.003	1.33	1.06–1.65	0.012
Transport method						
Self	Reference			Reference		
Ambulance	2.57	1.80–3.66	<0.001	2.98	2.02–4.41	<0.001
Reason for visit						
Injury/poisoning	Reference			Reference		
Disease	1.47	1.17–1.84	0.001	1.82	1.40–2.37	<0.001
Provided medical service						
Examination (only)	Reference			Reference		
Emergency medication/procedure	0.94	0.60–1.47	0.771	1.30	0.80–2.11	0.283
Operation	3.83	2.74–5.34	<0.001	5.40	3.74–7.81	<0.001

## Discussion

According to Kubicek et al. [10], demographic factors such as medical insurance coverage, type of insurance coverage, and low household income were closely related to ED visit frequency and severity. Although previous studies in the US evaluated individual and household demographic characteristics of pediatric ED patients [14], previous reports on pediatric ED patients in Korea have focused mainly on clinical characteristics [3,15]. To the best of our knowledge, our study is the first to explore both clinical and demographic characteristics with regard to ED use among the Korean pediatric population.

In terms of sex and age group, our study found that males made more ED visits than females and younger children (age 0–5) made more visits than older children. These findings are similar to those of Kwak et al. [3] in Korea and Ben-Isaac et al. [14] in the US. Korean childrenin the two highest quintiles of household income (49.2%) visited the ED more often than those in the two lowest quintiles (24.7%), perhaps due to the relatively high cost of ED services. This trend was also observed in the US, where middle- and upper- income (≥ 200% of federal poverty level, 56.1%) children made more ED visits than children from poor households (≤ 124% of federal poverty level, 27.5%) [14].

In our study, the rate of ambulance transport (5.2%) was similar to that reported by Kwak et al. [3] (5.3%), and our findings for visit reason (disease being 2.1 times more common than injury/poisoning) closely matched those of Kwak et al. [3], who reported disease as being 2.5 times more common. We conducted post-hoc subgroup analysis to explore the difference in visit reason according to sex, and found a significant difference (p<0.001). ED visits for injury/poisoning were 1.8 times greater in males than in females, whereas ED visits for disease were only 1.2 times greater in males than in females. This discrepancy might be due to higher levels of curiosity and risk-taking among boys [16].

The odds of hospital admission were lower in females than males (OR = 0.73); this was similar to the report of Kwak et al. [3], in which the odds for males were 1.11 times higher than odds for females. The odds of hospital admission for children living in the capital were lower than for those living in provinces. We speculate that relatively high accessibility to the ED enabled capital-dwelling patients to visit the ED more frequently even if their conditions did not necessitate admission. Children in the highest quintile of household income and children covered by private insurance had lower odds for hospital admission than those in the lowest income quintile and those lacking private insurance, respectively. Again, this might reflect the ability of children with high household income or private insurance to visit the ED more frequently, even for conditions not of requiring admission. Visits for disease had higher odds of hospital admission than visits for injury/poisoning, as was previously reported by Kwak et al. [3].

To establish policies for desirable changes to the emergency medical service system, comprehensive analysis of the current state of the system is required. In that respect, our study results can be used as baseline data for future research. For example, based on our finding that children with injury/poisoning had lower odds of hospital admission than children with disease, additional studies regarding children visiting the ED after injury/poisoning could be conducted to investigate whether it would be possible to lower admissions due to injury/poisoning further still. To do this, descriptive analysis of injured children with regard to more detailed injury characteristics and injury related outcomes would be required. In addition, interventional studies investigating the efficacy of strategies to reduce non-urgent ED visits (for example, public relations about first aid for frequent injuries) could be conducted.

One limitation of this study is the possibility of recall bias because the data were collected retrospectively. However, to minimize this bias, data were collected by trained investigators, and household accounts of health service usage were cross-referenced to receipts for medical costs for each panel household. Another limitation is that we only examined the association between characteristics of pediatric ED patients and hospital admission after ED treatment. We did not investigate criteria for distinguishing urgent versus non-urgent emergencies other than hospital admission. As a result, detailed analysis of patients who were admitted to the emergency room with regard to urgency was not possible. Lastly, given the retrospective study design, some possible confounding factors affecting hospital admission might have been missed.

In conclusion, the only medical service provided to the majority of pediatric ED patients was diagnostic examination, and only 14.3% of ED visits resulted in hospital admission. Male sex, residence in the capital, private insurance coverage, and disease as a reason for the ED visit were closely associated with hospital admission. Based on the results of this study, prospective interventional studies might be required to establish policies regarding appropriate and effective pediatric ED service in the future.
